# Neural Interactome: Interactive Simulation of a Neuronal System

**DOI:** 10.3389/fncom.2019.00008

**Published:** 2019-03-13

**Authors:** Jimin Kim, William Leahy, Eli Shlizerman

**Affiliations:** ^1^Department of Electrical and Computer Engineering, University of Washington, Seattle, WA, United States; ^2^Department of Applied Mathematics, University of Washington, Seattle, WA, United States

**Keywords:** *C. elegans*, brain simulation, connectome, neural dynamics, network visualization

## Abstract

Connectivity and biophysical processes determine the functionality of neuronal networks. We, therefore, developed a real-time framework, called Neural Interactome^1^^,^^2^, to simultaneously visualize and interact with the structure and dynamics of such networks. Neural Interactome is a cross-platform framework, which combines graph visualization with the simulation of neural dynamics, or experimentally recorded multi neural time series, to allow application of stimuli to neurons to examine network responses. In addition, Neural Interactome supports structural changes, such as disconnection of neurons from the network (ablation feature). Neural dynamics can be explored on a single neuron level (using a zoom feature), back in time (using a review feature), and recorded (using presets feature). The development of the Neural Interactome was guided by generic concepts to be applicable to neuronal networks with different neural connectivity and dynamics. We implement the framework using a model of the nervous system of *Caenorhabditis elegans* (*C. elegans*) nematode, a model organism with resolved connectome and neural dynamics. We show that Neural Interactome assists in studying neural response patterns associated with locomotion and other stimuli. In particular, we demonstrate how stimulation and ablation help in identifying neurons that shape particular dynamics. We examine scenarios that were experimentally studied, such as touch response circuit, and explore new scenarios that did not undergo elaborate experimental studies.

## Introduction

Modeling neuronal systems involves incorporating two modeling layers. The first fundamental layer is of neuronal connectivity (connectome). The layer on top of it is of biophysical processes of neural responses and interactions. In the recent years there has been significant progress in resolving and modeling both layers. Connectomes of several organisms and systems, such as the nematode *Caenorhabditis elegans (C. elegans)*, the *Drosophila* medulla, the mouse retina, mouse primary visual cortex, and others have been fully or partially mapped on various scales: from macro to single neuron level (White et al., 1986; Open Connectome Project, 2010; Van Den Heuvel and Pol, 2010; Bock et al., 2011; Briggman et al., 2011; Haspel and O'Donovan, 2011; Varshney et al., 2011). Also, decades of research in describing and modeling biophysical processes have provided both experimental and computational foundations for modeling single neuron dynamics as well as synaptic and electric processes between neurons (Koch and Segev, 1988; Wicks et al., 1996; Letinic et al., 2002; Koch, 2004; Söhl et al., 2005; Briggman et al., 2006; Skinner, 2012; Druckmann et al., 2014; Kunert et al., 2014). Due to these advances, models incorporating both layers have become more detailed and realizable for several neuronal systems. These models are called *Dynomes* as they correspond to dynamical system acting on top of the static connectome (Kopell et al., 2014).

Being closer to the realistic neuronal system, *dynome* studies have more potential to reveal neural pathways and functionalities of the network (Bargmann and Marder, 2013; Sporns and Bullmore, 2014; Liu et al., 2018). However, they also introduce challenges in finding appropriate methods for efficient studies of network capabilities (Mucha et al., 2010). Brute force approaches will typically produce formidable amounts of data, where extraction or characterization of relevant neural patterns can be cumbersome and time consuming. For that reason, collaborative initiatives such as Brian introduced generic simulation engines for neural dynamics and the OpenWorm project (incorporating Geppetto engine) suggested to apply generic neural models to *C. elegans* (Goodman and Brette, 2008, 2009; Raikov and De Schutter, 2012; Szigeti et al., 2014; Chen and De Schutter, 2017; Cantarelli et al., 2018; Sarma et al., 2018). Such frameworks are advantageous and allow flexibility to simulate various dynamics on top of generic connectomics.

Here, we have taken a complementary approach. We focus on *efficient* simulation of the *established* connectome of *C. elegans* somatic nervous system in conjunction with *established biophysical dynamics*. We have accompanied the simulation with interpretable visualization of dynamics-connectomics. The visualization is designed in such a way that it incorporates real-time interactive capabilities to investigate architecture and observe neural activity at the same time. Such a framework allows for a new way of investigating and simulating neuronal systems and as far as we know has not been introduced for any dynamic network, in particular nervous systems models. In such a framework, the necessary components are (i) ability to apply or modify stimuli to the network in real-time as in experiments; (ii) being able to observe the neural dynamics on various time and population scales, and (iii) allow for network structural changes. Furthermore, the framework is expected to perform seamless integration for such functions and include review capabilities for exploration of the system and dynamics in depth. In this work, we thereby develop the Neural Interactome, which is a generalized visualization framework incorporating such capabilities. The framework employs a graph visualization layout to represent the static connectome. On top of the layout, it incorporates dynamic visual components to represent real-time neural responses according to user interactions. These components are implemented via synchronization between the backend neural integrator of the *dynome* and the graph layout of the interactive interface. The backend neural integrator is connected to neurons stimuli panel, and permits setting external stimuli and changing the structure of the graph on demand. The framework also includes real-time plotting of neural activity as well as review, preset and save modes that allow for further exploration of simulated dynamics.

In this paper, we focus on applying the framework to the nervous system of *C. elegans* nematode, which consists of 302 neurons with three types (sensory, inter, motor). Such a system is thus relatively small to be fully reconstructed and analyzed. Indeed, the near-complete connectome of the nervous system has been resolved using serial section electron microscopy (White et al., 1986; Chen et al., 2006; Varshney et al., 2011). The connectome data includes enumeration of neural connections for the complete somatic nervous system (279 neurons) of *synaptic* type, where *GABAergic* neurons make inhibitory synapses, and *glutamergic* and *cholinergic* neurons form excitatory synapses. The connectome also enumerates *gap* junctions (electrical connections) for each pair of neurons. The connectome data is robust, since *C. elegans* neurons are recognizable and consistent throughout individual worms (White et al., 1986). Furthermore, *C. elegans* synaptic and gap connections are common across animals with more than 75% reproducibility (White et al., 1986; Durbin, 1987; Hall and Russell, 1991; Bargmann, 1993). In addition to the anatomical structure of the nervous system, biophysical *in-situ* recordings of membrane voltage response to input current injected into each individual neuron in the network have been performed (Wicks et al., 1996; Goodman et al., 2012). These revealed that *C. elegans* neurons are of non-spiking type with graded potential membrane voltage profile (Goodman et al., 1998). Following these studies, a set of mathematical models describing neural membrane voltage and interaction between the neurons were developed (Goodman et al., 1998; Kunert et al., 2014).

The availability of near-complete connectome data along with experimental quantification of responses and interactions provided a computational basis for reconstructing both static and dynamic layers of *C. elegans* neuronal network. Combination of these two layers was recently developed (Kunert et al., 2014). When applied with prescribed input stimuli, *C. elegans dynome* was capable of producing various forms of characteristic dynamics such as static, oscillatory, non-oscillatory and transient voltage patterns (Kunert-Graf et al., 2017). These dynamics indicated that *C. elegans dynome* is a valuable model for the worm's nervous system, and patterns observed are suggested to be consistent with the experimentally observed ones. In particular, stimulation of sensory PLM neurons with constant current resulted in a two-mode dominant oscillatory behavior in forward locomotion motor neurons (Kunert et al., 2014). The model is expected to include a variety of other additional patterns, however, their full validation is formidable to perform, as it requires many simulations with various stimuli amplitudes and combinations. For instance, in the context of touch response, it would be valuable to examine stimulation of ALM and AVM sensory neurons, which in experiments was identified as associated with anterior touch response and expressed as backward crawling (Chalfie et al., 1985; Driscoll and Kaplan, 1997). Furthermore, transitions from one type of dynamics to another (e.g., from oscillatory to non-oscillatory) are also expected to exist when input stimuli shift from one value to another. It is thereby introduction of a framework that facilitates these studies can assist in such goal.

## Design and Implementation

We first describe the main components of the Neural Interactome framework, and then continue to demonstrate its application to the nervous system of *C. elegans* worm for stimulation scenarios.

## Interactive Interface for Neuronal Network

The frontend of Neural Interactome is an interactive interface consisting of (i) neural stimulation/ablation interface, (ii) visualization of dynamics, (iii) control of simulation timescale, and (iv) review system.

### Neural Stimulation and Ablation

Neural stimuli are controlled by stimulation panel located on the left side of the screen. The panel enslists and categorizes all neurons in the network into three group types (sensory, inter, motor). Each group type is given a characteristic color (sensory: blue, inter: green, motor: red). Each individual neuron on the panel is a clickable button with a scrollable bar, which allows setting amplitudes of constant stimulus, i.e., inject current to the neuron (of nA nano-ampere unit). The amplitude of the stimulus can be adjusted prior to running a simulation (as initial condition), or at any time during the simulation. When stimulus is being adjusted during the simulation, it effectively imitates “clamping” of neurons in the network. In addition, to allow for testing various structural configurations for the network, the panel is designed to support neural ablation of neurons. By clicking on a neuron while holding the shift key, the neuron is grayed-out in the interface. Such operation disconnects the neuron from all of its respective connections (both receiving and outgoing) in both synaptic and gap type and thus effectively removes it from the network. The ablation can also be undone (reinsertion) by repeating the operation of shift key + clicking on the ablated neuron. Similar to neural stimulation, both ablation and reinsertion can be performed prior and during network simulation.

### Dynome Visualization

#### Connectivity Representation

**Figure 1:**
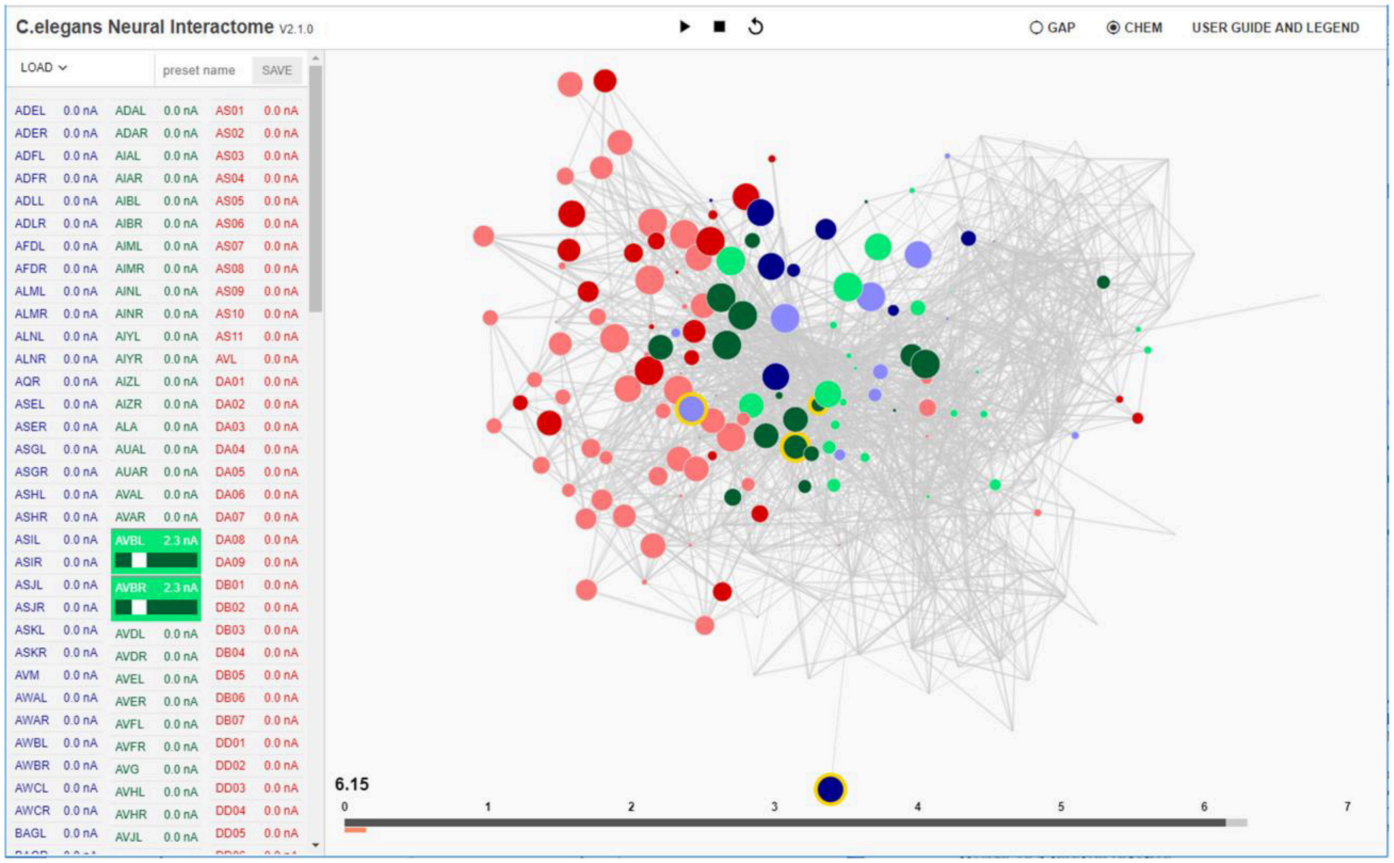
Interactive interface for Neural Interactome. Left panel enlists all the neurons classified by type (sensory, inter and motor). Each neuron is a clickable button with a scroll option. Scrolling adjusts the magnitude of constant stimulus; shift + click ablates the neuron from the network. Right: Force-directed graph displays each neuron's membrane voltage (node color denotes the sign; radius denotes the magnitude) and connections between neurons (edges between each pair of nodes). At the bottom of the graph, time bar keeps track of visualized time point (dark gray), and of computed time by the backend neural integration (light gray).

**Figure 2:**
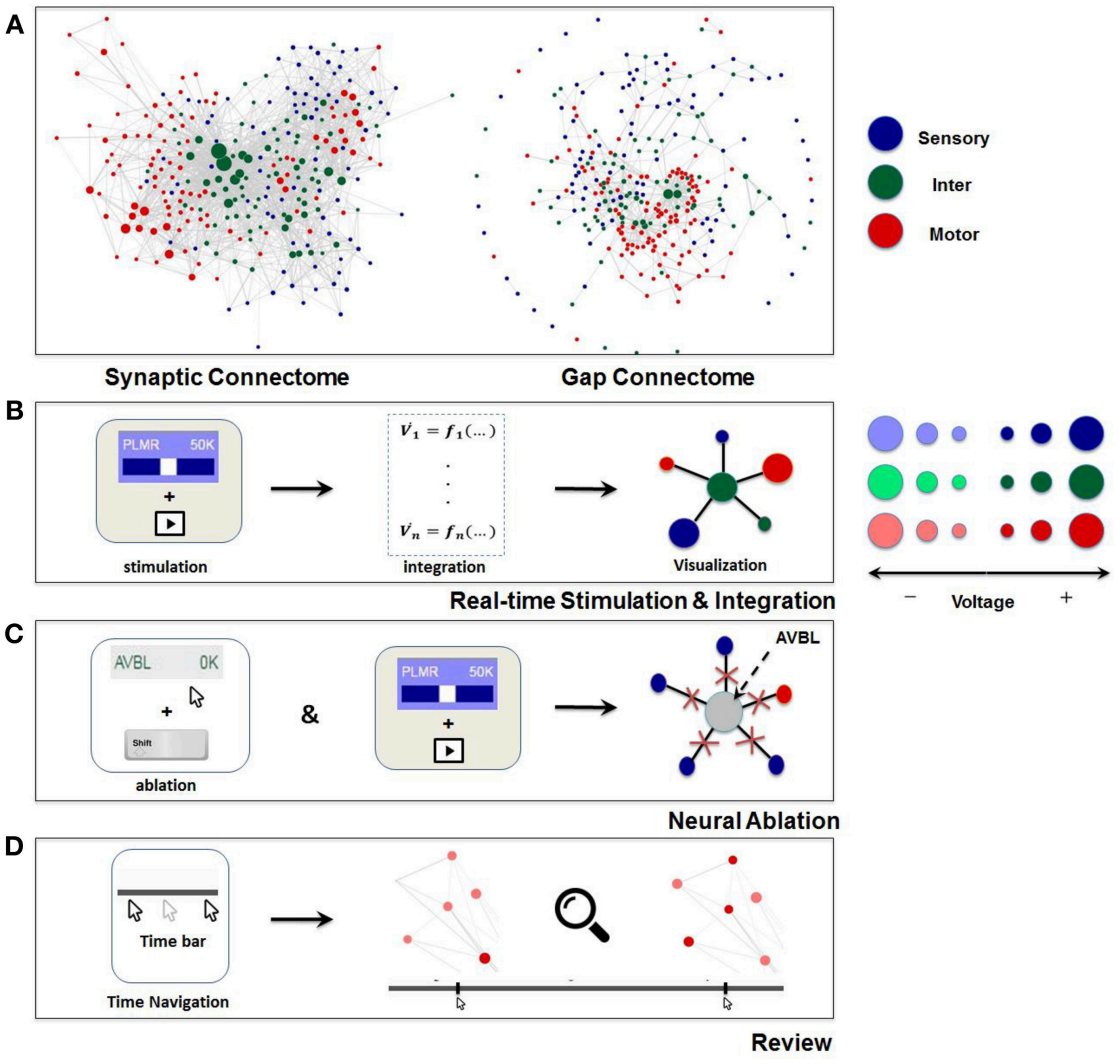
Visualization and main functionalities of Neural Interactome. **(A)** Force-directed graph visualization of *C. elegans* worm's synaptic (chemical, left) and gap (electrical, right) connectomes. Each node represents individual neuron colored according to its group type and edges represent connections (either synaptic or gap). **(B)** Schematics of Neural Interactome real-time stimulation and integration component; when user stimulates a neuron with the interface, the backend integrator computes membrane voltages in response to the stimuli, which are then visualized on the graph in respect to their signs and magnitudes. **(C)** Neural ablation is performed by clicking on the neuron, while holding the shift key. Ablation disconnects all the connections of the neuron (both gap and synaptic). **(D)** The review system implements a clickable time bar to allow navigation to any previously computed time point in the simulated dynamics. This is further enhanced with dynamics zoom-in/zoom-out feature designed for in-depth analysis of connectivity structure and local dynamics of sub-circuits.

Visualization of the *dynome* is on the right side of the interactive interface, with the connectome of the network represented as a graph (Figure 1). The nodes of the graph represent neurons, whereas the edges represent connections (either gap or synaptic) between each pair of neurons. The top panel of Figure 2 shows *C. elegans'* synaptic connectome (left) as well as its gap connectome (right), where each node represents an individual neuron and colored according to its group type. Initially, prior to displaying the *dynome* dynamics, the radii of the nodes are set according to in/out synaptic degree of the respective neuron (i.e., the amount of synaptic connections of a neuron). Such visualization emphasizes neurons with higher degree (hub neurons) by displaying them with larger radius and de-emphasizes neurons with lower degree with smaller radius. The width of the edge between a pair of neurons is set according to maximum synaptic weight, such that for a pair of neurons A and B, width_edge(A, B)_ = *max*(n_Syn(A → B)_, n_Syn(B → A)_), where *n*_*Syn*(*A*→*B*)_ is the number of synapses from neuron A to B.

In addition, we use force-directed graph algorithm to arrange the nodes and edges in optimal positions (Bostock et al., 2011). The algorithm visualizes graphs by assigning forces to nodes and edges based on their relative positions and routings. For edges, spring-like attractive forces based on Hooke's law are used to attract pairs of endpoints toward each other. For the nodes, repulsive forces, e.g., Coulomb's law forces, are used to separate all pairs of nodes. Once forces are assigned, the algorithm minimizes the total energy potential of the system (i.e., equilibrium states for the system of forces) and displays optimal nodes and edges configuration. In this representation, the edges tend to have uniform lengths (due to spring forces) and the nodes not connected by an edge tend to be drawn further apart (due to repulsive forces) (Kobourov, 2012). We found such graph visualization more advantageous for neuronal networks than the anatomy based visualization method as it: (i) keeps approximately equal lengths for all neuron's connections thus avoiding “clumps” of neurons in one region, and (ii) arranges the nodes such that neurons that make connections with a particular neuron are found within its proximity. We also found that due to these properties, the configuration depicts the network in an intuitive way, by grouping the same type of neurons together (e.g., many of the motor neurons are clustered together on the left of the graph) and places the neurons with high synaptic degrees in the middle. To keep the same frame of reference, force-directed representation is pre-computed before the simulation such that the positions of the nodes remain constant at all times.

#### Neural Activity Visualization

Neural activity is represented as an additional layer on top of the static connectivity graph. We find that optimal approach to visualize the two layers is to alter graph components (nodes and edges) according to neural activity. This creates a “breathing graph” which represents network activity and structure in real-time. In particular, we propose dynamic change to radii and colors of the nodes to depict neural activity. Changes are typically noticeable when the visualized variable representing the activity is continuous and scaled. In addition, it is beneficial that the visualized variable will have a continuous and interpretable meaning. Notable candidates for such variables are SR calcium activation dynamics or instantaneous firing rates (*peri stimulus time histograms* PSTH) (Palm et al., 1988; Schatzmann, 1989; Egelman and Montague, 1999). SR calcium activation is a scalable continuous process representing transformation of membrane voltage dynamics (spiking, bursts separated by near-silent interburst periods, and graded voltage potentials) to an activation variable. It serves as a vital biophysical signal associated with activation of muscle activity (McMillen and Holmes, 2006). In addition, several recording techniques quantifying neural dynamics are capable to measure and monitor SR calcium activity and can be directly compared with the visualization. The PSTH variable is computed from spiking dynamics and represents a spike count over a sliding window in time (Dayan and Abbott, 2001). Such a measure is applied to both measurements of spiking membrane voltage or a computational model that produces spike trains. PSTH is a continuous and scaled measure widely used for identification, classification and recognition of response patterns associated with stimuli (Riffell et al., 2014; Shlizerman et al., 2014).

To visualize these activity variables we propose to alter the radius and the color of the nodes. When the variable is a signed number (as in SR calcium activation) we use the radius to represent variable's amplitude and assign a color map to represent its sign. When the variable is unsigned, as in the case of PSTH, only one node component (either color or radius) is needed to represent its amplitude, and the other component can be utilized for visualization of additional information such as spike times. For example, when the radius is used to depict the PSTH amplitude, color flickering can be used to display the occurrence of spikes.

For *C. elegans* network we transform membrane voltage to SR calcium like activation variable to represent neural dynamics. In particular, membrane voltages, computed by backend neural integrator, described further in “*Backend Neural Integration*”, are translated to the following metric of radius size according to:

(1) $$\begin{array}{r} |R_{i}|=\frac{R_{\max}|V_{i}{|}^{2}}{\rho +|V_{i}{|}^{2}}\\ \text{sign }\left({\text{R}}_{\text{i}}\right)=\text{sign}\left({\text{V}}_{\text{i}}\right) \end{array}$$

where *R*_max_ is the maximum radii of the nodes and ρ is the slope factor. The sign of *R*_*i*_ is determined by the sign of the voltage *V*_*i*_. Such scaling of membrane voltages allows discerning active neurons at each given time without having to visualize the raw voltages. While in *C. elegans* membrane voltages are graded potentials, similar scaling accommodates other diverse types of neural activity, e.g., bursts, oscillations, etc (Rahmati et al., 2016).

Observing the colors and radii scaling over time allows to visually capture the unique patterns of dynamics on a population level, specifically oscillations, sudden bursts, settling down of dynamics. For example, when a population of neurons exhibit oscillations, colors will distinguish representatives of particular groups that are active and dynamically change their tones to display the fluctuation between positive and negative voltages. Indeed, for *C. elegans* network we show how we can identify oscillatory sub circuits of motor neurons, which fluctuate from negative to positive values over the period of 2 sec, upon stimulation of PLM touch sensitive sensory neurons. To further aid the investigation, the interface displays a plot of neuron's membrane voltage over time when the user hovers over a node.

#### Simulation Timescale

We implement the simulation timescale to be typically slower than the actual time in order to: (i) balance computations performed by the backend, and (ii) allow users to capture the details of the dynamics, as visualization in actual timescale tends to happen quickly. We also design the timescales of the stimulations to be dynamic, such that during stimuli transition or neural ablation, running time temporarily slows down to capture the dynamics that occur during the transition.

On the bottom of the interface we locate the time bar, which serves as the interface to interact with the timescales of the visualization (Figure 1). It consists of two bars; the dark gray bar shows the current time in visualization, while the light gray bar shows the computed time by the backend. We have developed the time bar to be similar to a streaming bar, which is widely implemented in popular video-hosting websites such as YouTube, and provides the interface to our *review system*, as we describe next.

### Review System

The *review system* allows for isolating various time and population scales for further analysis (Figures 2D). Using the time bar, we add the ability to navigate back to any previously computed time by clicking on a desired time point within the time bar (analogous to navigating back and forth while playing a video). In such a case the network along with the dark gray bar are set to the state at the selected time point. Time navigation can be done either during simulation or when simulation is paused. In the former, the simulation will continue onward from the selected time point while for the latter, it will display the paused dynamics at that time point. In addition, we assign left and right arrow keys on the keyboard to control visualization speed (Fast FWD and Fast BWD). When activated during simulation or paused, the left and right arrow keys increase visualization speed while browsing through the dynamics in both directions.

An additional component of the *review system* is the dynamic zoom-in/out feature, which focuses into sub circuits within the network at any time during the simulation. It is implemented by uniformly scaling the lengths of the edges and keeping the nodes radii the same. Effectively, such a method is optimal for observing a small group of neurons, as it increases the spacing between nodes and displays local sub circuit connectivity structure and dynamics (Figure 2D). Hovering with a mouse over a neuron will also highlight the connections it makes to neighbor neurons, and will display their labels categorized in different group type colors.

In addition, features such as “presets” and “save dynamics” are implemented as part of the review system. *Presets* allow users to save configurations of neurons stimuli panel whereas *save dynamics* stores the voltage time series data for all neurons during a single session as a file. *Presets* can be used to save stimuli configuration, ablation configuration, or both, whereas *save dynamics* can be used to perform detailed analysis/comparison with the experiments against the simulated dynamics. To create a preset, one can enter the name of the preset above the neurons stimuli panel while the panel is configured to desired setup (Figure 1) and click SAVE button. Upon exiting or resetting the interface, *save dynamics* will automatically save the time series data in npy file format (compatible with Python NumPy library) in “saved_dynamics” folder within the software directory.

## Backend Neural Integration

Backend neural integration computes neural activity for the whole network for a time interval [*t, t*+Δ*t*] and transmits these values to the interactive interface for visualization. In *C. elegans*, the integrator is solving a system of non-linear ordinary differential equations with 558 dimensions (279 for neurons voltage and 279 for synaptic variables) that model the biophysical processes and interactions between neurons. Such high dimensional ODE system is not computationally trivial, thus we implement an efficient vectorized adaptive solution. Specifically, the following equations are being integrated (see Kunert et al., 2014 for more details):

(2) $$\begin{array}{r} \text{C}\frac{{\text{dV}}_{\text{i}}}{\text{dt}}=-{\text{G}}^{\text{c}}\left({\text{V}}_{\text{i}}-{\text{E}}_{\text{cell}}\right)-{\text{I}}_{\text{i}}^{\text{Gap}}\left(\text{Ṽ}\right)-{\text{I}}_{\text{i}}^{\text{Syn}}\left(\text{Ṽ}\right)+{\text{I}}_{\text{i}}^{\text{Ext}} \end{array}$$

(3) $$\begin{array}{r} {\text{I}}_{\text{i}}^{\text{Gap}}\left(\text{Ṽ}\right)=\underset{\text{j}}{\sum }{\text{G}}_{\text{ij}}^{\text{g}}\left({\text{V}}_{\text{i}}-{\text{V}}_{\text{j}}\right) \end{array}$$

(4) $$\begin{array}{r} {\text{I}}_{\text{i}}^{\text{Syn}}\left(\text{Ṽ}\right)=\underset{\text{j}}{\sum }{\text{G}}_{\text{ij}}^{\text{s}}{\text{s}}_{\text{j}}\left({\text{V}}_{\text{i}}-{\text{E}}_{\text{j}}\right) \end{array}$$

(5) $$\begin{array}{l} \frac{{\text{ds}}_{\text{i}}}{\text{dt}}={\text{a}}_{\text{r}}\Phi \left({\text{V}}_{\text{i}};\beta ,{\text{V}}_{\text{th}}\right)\left(1-{\text{s}}_{\text{i}}\right)-{\text{a}}_{\text{d}}{\text{s}}_{\text{i}} \end{array}$$

(6) $$\begin{array}{r} \Phi \left({\text{V}}_{\text{i}};\beta ,{\text{V}}_{\text{th}}\right)=\frac{1}{1+\text{exp}\left(-\beta \left({\text{V}}_{\text{i}}-{\text{V}}_{\text{th}}\right)\right)} \end{array}$$

Where **C** is the cell membrane capacitance, G^c^ is the cell membrane conductance, E_cell_ is the leakage potential, and ${\text{I}}_{\text{i}}^{\text{Gap}}\left(\text{Ṽ}\right)$, ${\text{I}}_{\text{i}}^{\text{Syn}}\left(\text{Ṽ}\right)$, and ${\text{I}}_{\text{i}}^{\text{Ext}}$ each correspond to input current contributed by gap junctions, synapses, and external input stimuli. ${\text{G}}_{\text{ij}}^{\text{g}}$ and ${\text{G}}_{\text{ij}}^{\text{s}}$ each correspond to total conductivity of gap junctions between i and j and maximum total conductivity of synapses to i from j, where ${\text{G}}_{\text{ij}}^{\text{s}}$ is modulated by synaptic activity variable s_i_. The synaptic activity variable is governed by Equation (5), where a_r_ and a_d_ correspond to the synaptic activity rise and decay time, and Φ is the sigmoid function with width β. The equations are based on *in-situ* recordings of membrane voltage indicating that neuron responses are graded potentials and hence better fit to describe the voltage dynamics than standard multi-compartmental spiking neural activity models.

While *C. elegans* neural activity is expressed through graded membrane potential, for other systems, especially systems in which neural activity is expressed through fast spiking, factors such as synaptic transmission delays due to finite propagation speeds and time lapses could appear and impact the network dynamics (Guo et al., 2012, 2016). Mathematically, such delays can be incorporated by introducing autaptic inhibition term I_aut(t) of form:

(7) $$\begin{array}{r} {\text{I}}_{\text{i}}^{\text{syn}}\left(\text{t}\right)=\underset{\text{j}}{\sum }{\text{g}}_{\text{ij}}^{\text{aut}}{\text{s}}_{\text{ij}}\left(\text{t}\right)\left({\text{E}}_{\text{syn}}-{\text{V}}_{\text{i}}\right) \end{array}$$

Where $g_{ij}^{aut}$ is the autaptic coupling strength from neuron j to neuron i and the corresponding synaptic variable s_ij(t) is described by identical first-order model as Equation. 5 with sigmoid function term Φ_d_ now including the transmission delay τ_d_ as follows:

(8) $$\begin{array}{r} \Phi_{\text{d}}=\frac{{\text{T}}_{\max}}{1+\text{exp}\left[-\beta_{\text{d}}\left({\text{V}}_{\text{j}}\left(\text{t}-\tau_{\text{d}}\right)-{\text{V}}_{\text{th}}\right)\right]} \end{array}$$

Where T_max_ is the maximal concentration of transmitter in the synaptic cleft, V_j_ is the pre-synaptic voltage. Since the computation of network activity is independent from the frontend visualization, the platform allows direct incorporation of such higher order effects.

### Synchronization of Integration and Visualization

To support real-time interaction, we implement a synchronization procedure through a communication system between the interface and the backend. Specifically, we use an object ODE integrator which supports event handling and adaptive time-stepping. This functionality allows us to establish a robust protocol between the interface and the backend to support interactive changes to the simulation parameters in real-time between solution points. The protocol monitors the following quantities: t_computed_: Computed time in the backend neural integration, t_visualization_: Visualized time in the interactive interface, Δ*t*: Data stack, i.e., time interval to be computed, t_buffer_: Buffer size between t_computed_ and t_visualization_, τ: Internal refractory period for checking t_computed_ − t_visualization_.

The system is implemented to keep t_computed_ − t_visualization_ ≅ t_buffer_ at all times such that backend neural integration is always responsive to real-time user interactions, but also accommodates computation of new solutions before the visualization fully catches up with the computation.

Based on these principles the communication protocol is as follows:

(i) The interface sends a command to the backend to compute solutions for the time interval of [t_computed_, t_computed_ + Δt] given the condition:

(9) $$\begin{array}{r} {\text{t}}_{\text{computed}}-{\text{t}}_{\text{visualization}}\leq {\text{t}}_{\text{buffer}}. \end{array}$$

(ii) Once the command has been sent, the interface waits for a new block of solution of size Δ*t* from the backend.

(iii) Once the block is received, the interface resumes to poll whether condition (9) is satisfied. Polling is performed as follows: If the condition is met, the system applies (i). If not, the system goes through a refractory period of τ and then checks again for condition (9).

**Figure 3:**
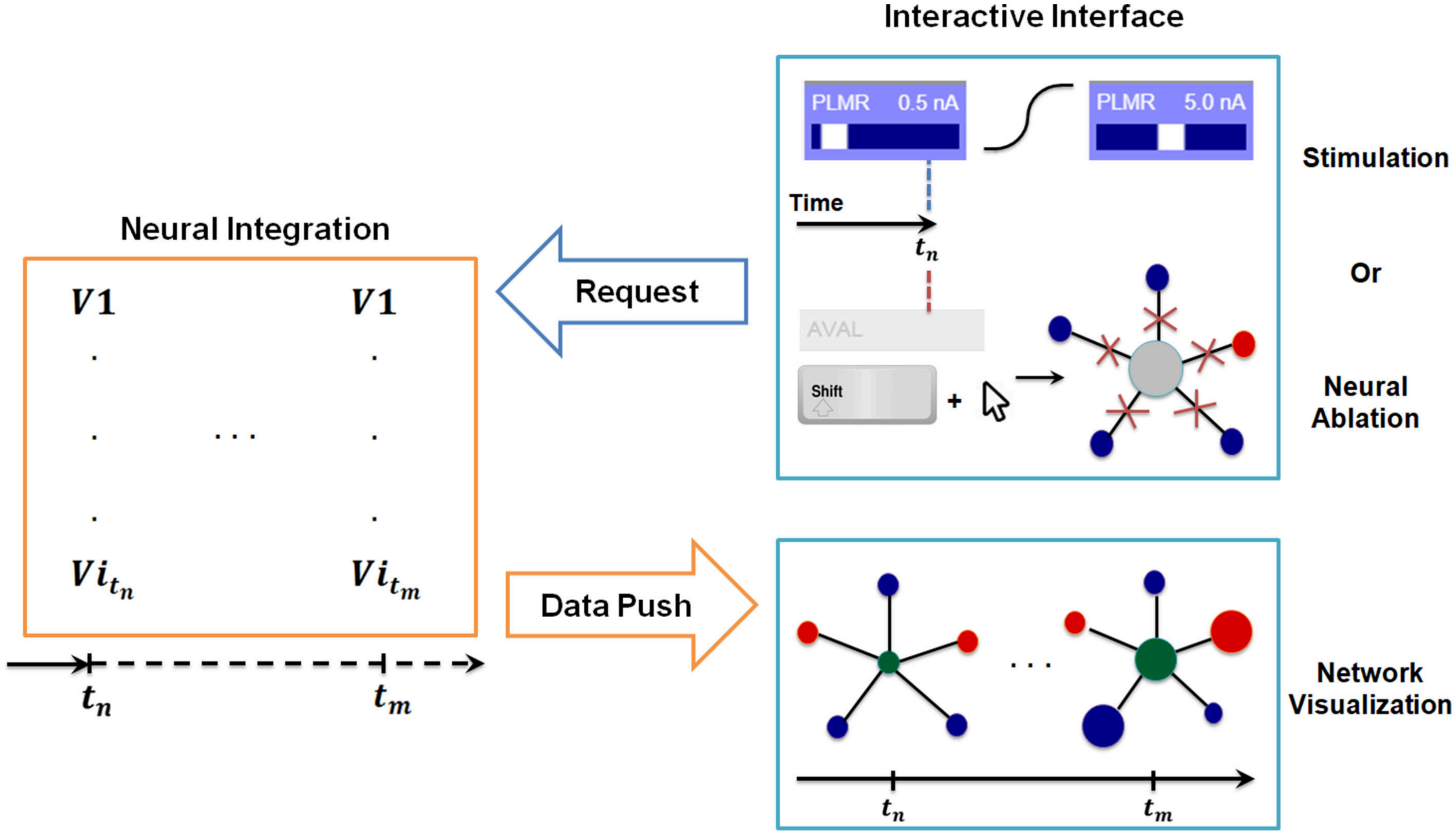
Synchronization between interactive interface and backend neural integration. The backend computes membrane voltage values for future time interval requested by the interface, and transmits them to the graph for visualization. User driven change in the interactive interface, i.e., stimulation or neural ablation, invokes a process that passes the information to the backend where relevant parameters of integration are modified.

In Figure 3 we include a diagram depicting how the synchronization method allows for stimulation of neurons at any given time and simultaneous inspection of network response to such actions. When the user stimulates a specific neuron (e.g., PLMR in Figure 3) or performs a neural ablation the interface sends a command to backend neural integration to modify necessary parameters. This is followed by an additional command from the interface to compute the solution for interval [t_stimulus_, t_stimulus_ + Δt]. The backend, upon receiving the first command, modifies the input stimuli parameters for stimulation or connectivity matrices for ablation. It then executes the second command by computing the voltage values for all neurons for a given time interval. The computed voltage values are then transmitted to the interface for visualization. This cycle of command and data transmission is repeated indefinitely until the simulation is stopped.

### Stimuli Transition

In addition to integration of the dynamical equations, the backend ensures that any modification of stimuli amplitude during stimulation is executed in a realistic manner (i.e., no sudden jumps or drops in the stimulus). Ensuring such continuity produces more realistic shift of stimuli from one value to the other. Explicitly, we determine the magnitude of stimulus during the transition through a combination of two hyperbolic tangent functions:

(10) $$\begin{array}{l} S_{transit}\left(\tau \right)={\text{S}}_{old}\left(\frac{1}{2}-\frac{1}{2}\tanh\left(\frac{t-\left(t_{switch}+t_{offset}\right)}{r}\right)\right)\\ \;+{\text{S}}_{new}\left(\frac{1}{2}+\frac{1}{2}\tanh\left(\frac{t-\left(t_{switch}+t_{offset}\right)}{r}\right)\right) \end{array}$$

Where *t*_*switch*_ is the time when the input current was modified, and *r*, *t*_*offset*_ are the constant coefficients that determine the width and initial point of the transition, respectively. Such construction makes sure that every transition takes place in a continuous manner and supports variable transition speeds of *r*.

### Neural Ablation

In addition, the backend implements neural ablation by instantaneous modification of connectivity matrices (both gap and synaptic). This step is followed by recalculation of the quantities in the network associated with the modified structure (e.g., the equilibrium states of the network Vth; see Materials and Methods section for more detail). Effectively, when the user ablates a neuron in the interface, an array that keeps track of active neurons (1-present, 0-ablated) is being updated. The modified array is then sent to the backend, where for each ablated neuron, say neuron *i*, all elements of the connectivity matrices in row and column *i* (corresponding to in/out connections) are set to zero.

Reinsertion of neurons after they were ablated implements the ablation operations in reverse order. Particularly, when the user reinserts the neuron, the interactive interface modifies the active neurons array, such that the corresponding neuron's entry is changed from 0 to 1. The modified array is then transmitted to the backend, where it will restore the corresponding row and column of the connectivity matrices to default values.

## Results

We proceed to demonstrate how application of Neural Interactome to *C. elegans* nervous system can assist in the study of neural dynamics. In particular, we target two sub circuits (i) a circuit associated with a touch response, which stimulation is known to be associated with forward and backward locomotion (ii) explore neural dynamic patterns induced by the excitation of sub group of sensory neurons, which recently were discovered to be associated with nictation behavior.

### Posterior Touch Response Stimulation Scenario

PLM sensory neurons (PLML/PLMR) in *C. elegans* nervous system are known as posterior mechanoreceptors. When stimulated by tail touch, PLM neurons excite motor neurons associated with forward crawling motion (Chalfie et al., 1985). AVB interneurons (AVBL/AVBR) are also known as driver cells for forward movement of the worm. We stimulate PLM sensory neurons and AVB interneurons with constant stimuli to examine neural patterns associated with forward crawling motion as a result of posterior touch response. We adjust the magnitudes of the input currents by scrolling stimuli bars in the interface. Specifically, we set 1.4 nA for PLM neurons, and 2.3 nA for AVB interneurons, which result in profound oscillations.

**Figure 4:**
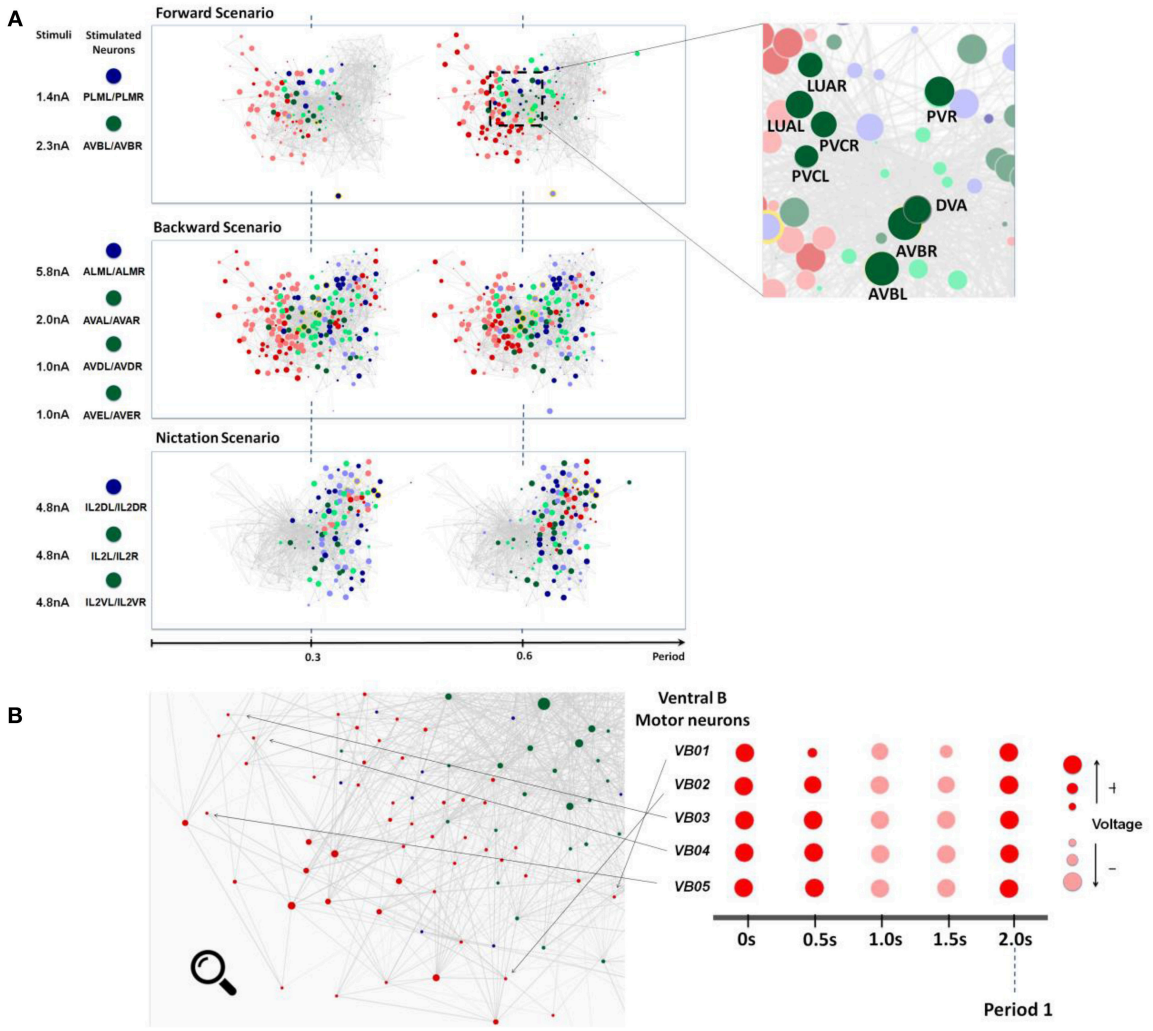
**(A)** Snapshots of neuronal responses corresponding to locomotion forward, backward, and nictation scenarios visualized by Neural Interactome. Forward scenario shows the snapshots of neural dynamics in response to PLM and AVB neurons stimulation. Each snapshot is taken at 30% and 60% into the average period of motor neurons oscillatory dynamics. On the right panel, the most responsive interneurons are highlighted. Backward scenario displays the snapshots of neural dynamics as a result of stimulation of ALM/AVA/AVD/AVE neurons. Nictation scenario displays the snapshots of dynamics upon stimulation of IL2 neurons. **(B)** Identification of unique oscillatory dynamics during forward scenario using the review mode. Visualization of motor-neurons sub circuit using the review system zoom-in function (left). Snapshots of Ventricular B motor neurons (VB01 ~ VB05) during forward scenario sampled five times with equal interval during 2 s periodic cycle (right).

As expected from experimental results and prior work, we observe oscillations in some populations of neurons. We therefore study their periodic cycle. In the top panel of Figure 4A, we show two snapshots of network dynamics taken at discrete percentages into the periodic cycle. We observe that the network graph responds with strong oscillation in about ~40% of the neurons with mostly motor neurons (marked in red) being specifically active.

We identify more detailed properties of the dynamics by inspecting the dynamic graph in review mode (Figure 4B). The interface allows us to identify most responsive neurons and classify them into different types. In motor neurons, most active neurons (by maximum voltage amplitude above the threshold) appear to be Ventricular and Dorsal type B (VB, DB) neurons alongside with Ventricular and Dorsal type D (VD, DD) and AS motorneurons (AS01 – AS10). These neurons have identical oscillatory period of ~2 s, however, their dynamics are out of phase to each other.

Most responsive interneurons turn out to be AVB, LUA, DVA, PVR, and PVC (Figure 4A). Indeed AVB, DVA, and PVC were experimentally shown to act as modulators for forward locomotion (Chalfie et al., 1985; Wicks et al., 1996; Driscoll and Kaplan, 1997). Notably, Neural Interactome also identifies relatively strong responses in LUA and PVR neurons. While these neurons have structural connections to PLM (LUA neurons are suggested to connect between PLM touch receptors, and PVR have gap junctions to PLM), their direct relation to forward locomotion was not affirmed (e.g., laser ablation of LUA did not lead to abnormalities of movement). Our analysis, however, suggests that these neurons are actively participating in the oscillations. These findings suggest that Neural Interactome can help find candidates of neurons correlated to particular dynamics, even for known sub circuits.

### Anterior Touch Response Stimulation Scenario

ALM neurons sense touch to the anterior body region (i.e., frontal body) and induce motor neurons dynamics associated with backward locomotion (Chalfie et al., 1985). Aiding this process are the AVA, AVD, and AVE interneurons which act as modulators for the motion. We therefore stimulate these neurons with input currents that lead to profound dynamics, in particular: ALM = 5.8 nA, AVA = 2.0 nA, and AVD/AVE = 1.0 nA.

The snapshots of network dynamics while stimulating these neurons are shown in the middle panel of Figure 4A and labeled as Backward Scenario. Notably, comparing forward vs. backward neural responses, the dynamics for backward responses involve much larger number of neurons (~90%) than that of forward responses. We find the most responsive motor neurons to be Ventricular Dorsal type A (VA, DA), Ventricular Dorsal type D (VD, DD) and AS (AS01-AS10). The oscillation behavior for each of these groups is of different phase, but their periods appear to be uniform around ~3.5 s. The results are consistent with the experimental observations which reported the A-type and D-type motor neurons coordinating the backward motion (Chalfie et al., 1985; Chalfie and White, 1988).

Zooming into particular populations of motor neurons we observe that individual motor neurons exhibit more complex and irregular patterns than those of the forward stimulation. Unlike the oscillations observed in forward stimulation which are characterized by predominantly smooth sinusoidal form, here motor neurons appear to have oscillatory patterns with various waveforms: some motor neurons repeat steep fluctuations between negative and positive voltage while some exhibit triangular type oscillations above their thresholds.

We also observe more activity within the interneurons. Most prominent ones appear to be AVA, AVDR, AVE, PVR, DVA, ADA, and SABV. Some of these neurons are indeed identified in the literature, AVA, AVD, AVE are characterized to act as modulators for backward motion and DVA is characterized to maintain activity (Chalfie et al., 1985; Wicks et al., 1996; Driscoll and Kaplan, 1997; Gray et al., 2005). However, we also find high activity in neurons such as PVR, ADA, and SABV. While PVR makes gap junction with ALM, its role in backward locomotion has not been yet clarified. For both ADA and SABV, their functionality has not been fully specified yet. As in the posterior touch response scenario, the discovery of these additional neurons participating in dynamics provides new insights regarding the neurosensory integration of anterior touch response behavior.

### IL2 Neurons Stimulation Scenario

It has been recently shown that IL2 neurons regulate the *nictation* behavior in which a worm stands on its tail and waves its head. Such behavior is known to be observed within the *dauer* larva (i.e., developmental stage nematode worms) to transport itself via hosts such as flies or birds (Lee et al., 2012). For non-*dauers*, targeted activation of IL2 neurons does not induce nictation possibly because IL2 neurons undergo a significant structural change at the *dauer* stage. In this scenario, we stimulate IL2 neurons through Neural Interactome to investigate motor neuron dynamics possibly linked to such behavior or its remnant.

We present snapshots of network dynamics induced by IL2 (IL2DL/IL2DR, IL2L/IL2R, IL2VL/IL2VR) neurons stimulation in the bottom panel of Figure 4A. Notably, the network activates neurons located mostly on right side of the graph. This is a different pattern than forward and backward patterns. Most responsive motor neurons for such stimulation are RMG, RMH, and RMED along with moderate responses within SMD and RMEL/RMER motor neurons. The oscillatory periods for these neurons are uniform around ~5.7 s with different phases. Particularly, RMHL and RMHR neurons each produce oscillations nearly anti-phase to each other. For RMG neurons, the oscillation wave of RMGL always preceded that of RMGR, suggesting phase displacement between oscillations of these two neurons. Oscillations among four SMD motor neurons (SMDDL, SMDDR, SMDVL, and SMDVR) as well as of three RME motor neurons (RMEL, RMER, RMED) were observed to be approximately in phase.

In the literature, these motor neurons are known to be involved with control of head muscles. RMG and RMH motor neurons innervate lateral four rows of head muscles while RME neurons innervate all eight rows of head muscles (White et al., 1986). SMD motor neurons are also known to innervate head muscles involved with search behaviors such as omega-shaped turns under absence of food in the environment (Gray et al., 2005). Remarkably, Neural Interactome shows no response among the motor neurons associated with forward/backward locomotion (such as Ventricular Dorsal A, B and D) and only shows response of neurons modulating head muscles. Such results suggest that the activation of IL2 neurons leads to periodic head movements with absence of locomotory behavior in the rest of the body. While this does not necessarily imply that such motor neurons pattern is linked to nictation, these observations provide particular hypotheses and insights about the relatively unknown sub circuit for further empirical studies.

## Scenarios: Ablation

To validate Neural Interactome's application to investigation of network structural changes, we perform two ablations in conjunction with previously performed scenarios. In particular, we remove AVB and AVA interneurons from the network and repeat the posterior touch response scenario to observe their effects on the dynamics.

### AVB Ablation

**Figure 5:**
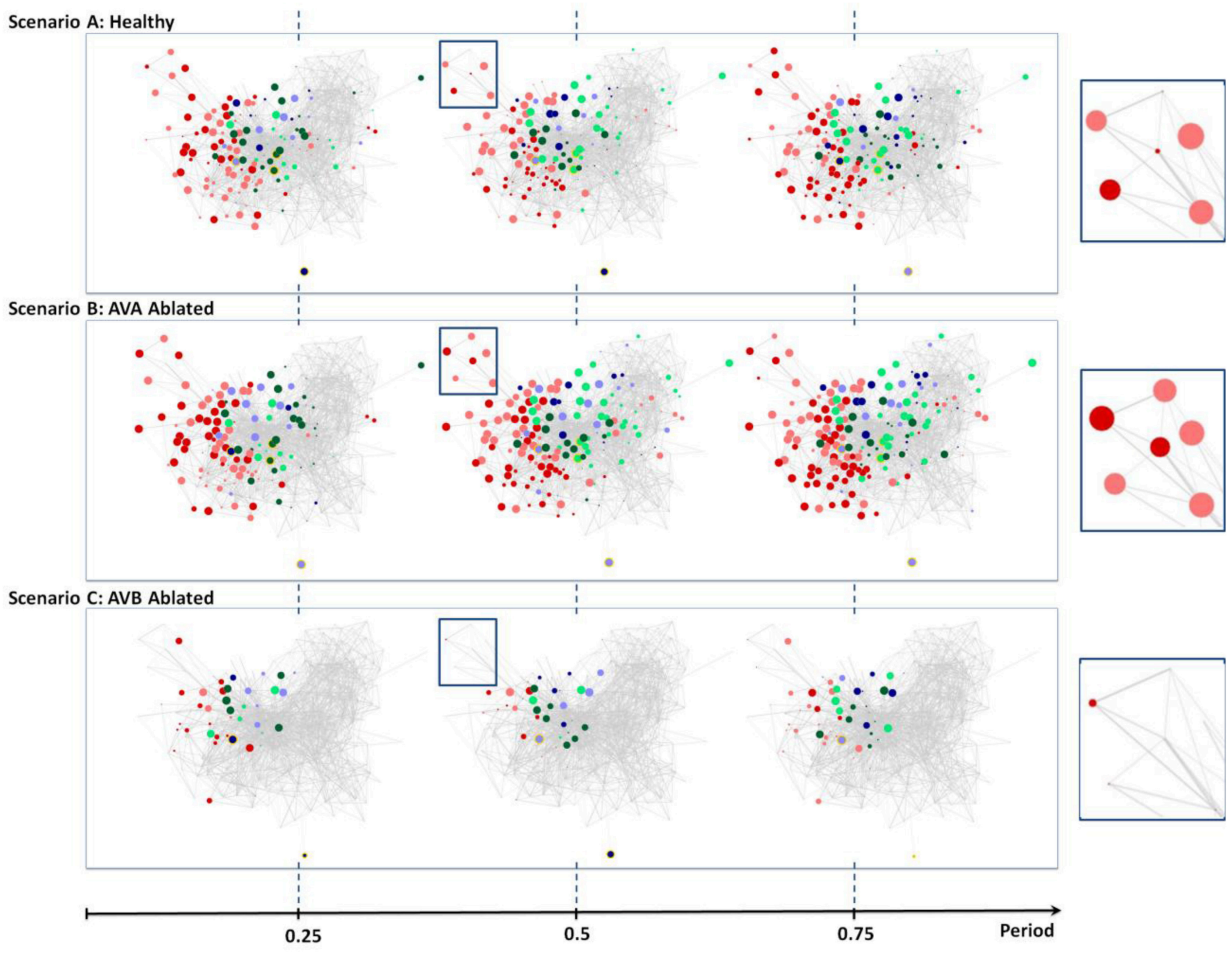
Neural dynamics during forward scenario for different structures of the network (non-ablated and ablated neurons). **(A)** Three snapshots of dynamics with healthy network (i.e., no ablation). **(B)** Snapshots of dynamics when AVA inter neurons are ablated. **(C)** Snapshots of dynamics when AVB inter neurons are ablated. All snapshots are taken at 25%, 50%, and 75% into each dynamics' oscillation period.

According to the literature, the removal of AVB neurons impedes forward locomotion (Chalfie et al., 1985). Indeed, we are able to confirm these experimental findings using Neural Interactome. Scenario C in Figure 5 shows the three snapshots of full periodic cycle upon repeating the posterior touch response scenario with AVB neurons ablated. We observe that neural patterns involve far less neurons than that of a healthy network (Figure 5, Scenario A). In particular, examination of full network snapshots as well as of local groups of motor neurons shows considerably weaker responses in comparison to the healthy structure.

The visualization does capture weak oscillations within a small group of motor neurons; particularly in Ventricular Dorsal B (VB, DB). Oscillation amplitudes are far less than the healthy dynamics, however, they remain to be relatively in phase and maintain an oscillatory period of ~1.9 s. We are unable to find any oscillatory activity within Ventricular Dorsal type D (VD, DD) neurons, which were out of phase with the activity of B type (VB, DB) neurons in the healthy case. Acknowledging that the two oscillatory phases property is necessary for the worm to perform forward crawling motion (Stephens et al., 2008), such observation confirms the experimental findings that the ablation of AVB neurons hinders the worm's ability to perform forward motion.

### AVA Ablation

Unlike the removal of AVB interneurons, experiments showed that the removal of AVA interneurons does not impact forward motion (Chalfie et al., 1985). Scenario B in Figure 5 shows snapshots of posterior touch response scenario with AVA neurons ablated. It is interesting to observe that the dynamics have slightly longer oscillatory period of about ~2.6 s. However, aside from that, the visualization shows that almost identical sets of neurons are active as in the healthy scenario (compare with Figure 4, Scenario A). We are also able to confirm, using the review mode, that the dynamics continue to exhibit strong oscillations in (VB, DB) & (VD, DD) motor neurons, with (VD, DD) neurons being out of phase to (VB, DB) neurons. Thus, our results for AVA ablation are consistent with experimental data in the literature.

Taken together our results show that Neural Interactome assists in confirming empirical results reported in the literature, and provides further insights regarding structure and activity associated with examined responses.

## Discussion

In this paper, we present a new visual interactive method, which we call Neural Interactome for studying the dynamics and the structure of a neuronal system (*dynome*). While it is important to simulate the full *dynome* to study network functionalities, multiple simulations of the *dynome* are formidable due to complexity in number of neurons and variations of stimuli. Neural Interactome approaches the problem through interactive real-time interface to the *dynome* and therefore significantly simplifies these studies. In particular, we show the simplicity of stimulating and ablating various groups of neurons in the framework.

To elucidate the overall structure and functionalities of the framework, we first define key components: (i) The interactive interface and (ii) the backend neural integration. Next, we apply it to *C. elegans* nematode, which connectome is resolved and the computational model describing both biophysical processes and interactions between neurons has been developed. We show that the framework provides novel possibilities to explore the worm's network structure and its unique neural patterns subject to stimuli. In particular, we demonstrate the Neural Interactome's capabilities using stimulations associated with the touch response: stimulation of PLM/AVB neurons for posterior touch, ALM/AVA/AVD/AVE neurons for anterior touch, and stimulation associated with nictation behavior: stimulation of IL2 sensory neurons. In all three scenarios, we observe clear visual characteristics of the induced neural patterns. For example, using the review features, we are able to identify most responsive neurons and additional properties of dynamics such as oscillation period and phase on individual and population level. By comparing such observations with behavioral and neural descriptions in the literature, we demonstrate that our results are consistent with the empirical observations of *C. elegans* locomotion and that they suggest additional novel insights.

In addition, we demonstrate the effectiveness and usability of the neural ablation feature in Neural Interactome by ablating hub interneurons (AVA or AVB). AVB ablation leads to network visualization with diminished activity in motor neurons as well as absence of characteristic out of phase oscillatory property required for such motion. The ablation of AVA interneurons, however, shows almost identical set of participating neurons as of the healthy network. We therefore believe that the framework has a potential to reveal other functionalities through multiple ablation scenarios, and provide further insights describing the role of the ablated neurons (Carrillo et al., 2013). In experiments, preparation and execution of ablation consumes significant time and usually requires special equipment, e.g., optogenetics. On the contrary, Neural Interactome can produce initial analyses for numerous ablation scenarios within seconds and consequently can be utilized as a pre-experiment tool to map scenarios for empirical exploration.

We designed the Neural Interactome to permit updates to both connectivity and dynamic models within the framework as they are further being refined in the future. Connectivity updates will merely require a change in the connectivity matrices. Replacement of a current model with more detailed one or different models (e.g., H-H type model) would merely require the replacement of the model itself, while the synchronization method between the neural neural integration and the interface will ensure that the computed values will be visualized properly. With such flexibility we expect that the framework will be similarly applicable to other neuronal systems: ranging from actual biological networks (such as that of *Drosophilla* medulla, the mouse retina, the mouse primary visual cortex) to artificial dynamic neural networks (e.g., Recurrent Neural Networks) and genetic networks (Alter, 2007). We also plan to keep adding more features to the framework to provide additional interaction possibilities with more detailed properties of the network, such as modification of individual synaptic or gap connections between a pair of neurons and more visualization options, such as plotting the comparison between multiple neuronal voltage dynamics. In addition to the functional features, we plan to incorporate more advanced computation methods such as parallel (GPU) computation for larger and more complex networks. The current simulation scheme is based on sequential time-stepping and supports event handling from user interactions. For network with moderate dimensionality such as *C. elegans* network, the overhead from incorporation of parallel computing for a single time step (i.e., GPU) and synchronization of the solution exceeds the time of solving it sequentially with CPU. However, for very large networks the single step computation efficiency could be of greater importance.

Beyond the simulation of *C. elegans* nervous system, we also plan to connect the model to musculature/body movement as they are critical components for model validation and for the study of interaction between neural dynamics and behavior. Development of such a model and connection of it with the *C. elegans* neural interactome could help in understanding how the neuronal network translates neural activity into behavior.

Neural Interactome can be either downloaded from Github repository or accessed online via a web interface with following addresses:

**Github**: https://github.com/shlizee/C-elegans-Neural-Interactome

**Web Interface**: http://neuralcode.amath.washington.edu/neuralinteractome

## Materials and Methods

In this section, we describe the materials and the methods used for the development of Neural Interactome and its application to *C. elegans* nervous system. The source code of the software is available at Github repository (https://github.com/shlizee/C-elegans-Neural-Interactome).

### Development Environment and Tools

We used two different programming languages for the development of Neural Interactome. We used Python to develop the backend neural integration, and Javascript to develop the frontend interactive interface. For establishing communication protocols between the interface and backend, we used flask-socketIO on Python side and Socket.IO on javascript side. Both flask-socketIO and Socket.IO are libraries that allow real-time bi-directional communication between the client (frontend) and the server (backend) through WebSocket protocols. In the context of Neural Interactome, they were used to establish robust command and data transactions between the interactive interface and backend neural integration.

Several third party libraries were used for each language as well. For Python, NumPy was used for mathematical computations and manipulations of matrices. Several functions from SciPy were used to construct the ordinary differential equation solver and solve the system of linear equations for computation of neural quantities such as V_threshold_ values.

In Javascript, D3.js (Data-driven documents) platform was used to construct force-directed graph representation of neuronal network. For the main webpage development framework, we used AngularJS as it provides optimal functionalities for building dynamic, single page web apps (SPAs).

### Threshold Potential (V_threshold_) Computation

Threshold potential for each neuron is computed by imposing $\frac{{\text{dV}}_{\text{i}}}{\text{dt}}=0$ (Equation 2 for *C. elegans*) and solving for V_i_. This is equivalent to Solving the following system of linear equations

(11) $$\begin{array}{r} \text{Ax}=\text{b} \end{array}$$

(12) $$\begin{array}{r} \text{A}={\text{M}}_{1}+{\text{M}}_{2}+{\text{M}}_{3};\text{b}=-{\text{b}}_{1}-{\text{b}}_{3}-{\text{I}}_{\text{ext}}, \end{array}$$

where the solution x is *N* × 1 vector with each entry being the threshold potential V_threshold_ for the i_th_ neuron.

M_1_ is a matrix of size *N* × *N* where *N* is the number of neurons (279 for *C. elegans*) with its diagonal terms populated with −G^c^ (cell membrane capacitance).

M_2_ is a diagonal matrix where diagonal term in i_th_ row corresponds to $-\underset{\text{j}}{\sum }{\text{G}}_{\text{ij}}^{\text{g}}$ i.e., the sum of total conductivity of gap junctions for the i_th_ neuron.

M_3_ is a diagonal matrix where its i_th_ diagonal term corresponds to $-\underset{\text{j}}{\sum }{\text{s}}_{\text{eq}}{\text{G}}_{\text{ij}}^{\text{s}}$, where ${\text{s}}_{\text{eq}}=\frac{{\text{a}}_{\text{r}}}{{\text{a}}_{\text{r}}+2{\text{a}}_{\text{d}}}$ and ${\text{G}}_{\text{ij}}^{\text{s}}$ is maximum total conductivity of synapses to i from j. Note that s_eq_ is obtained by imposing $\frac{{\text{ds}}_{\text{i}}}{\text{dt}}=0$ and synaptic activation Φ = 1/2 in Equation 5.

${\text{b}}_{1}={\text{G}}^{\text{c}*}{\text{E}}_{\text{c}}$ where E_c_ is a 1D vector of size *N* × 1 in which all of its elements are *E*_*c*_ (leakage potential).

${\text{b}}_{3}={\text{G}}^{\text{s}}\cdot \left({\text{s}}_{\text{eq}}^{*}{\text{E}}_{\text{j}}\right)$ where E_j_ is a 1D vector of size *N* × 1 that enlists the directionality of each neuron (0 if excitatory or −48 mV if inhibitory).

I_ext_ is the input stimuli vector where its i_th_ element determines the input current amplitude for the i_th_ neuron.

### Parameters

#### Dynome Visualization

We used *Rmax* = 15 as the maximum radius of the nodes in Equation 1.

#### ODEs Integration Parameters

The following values were used for relative/absolute tolerance and minimum integration step size. Note that the step size is determined adaptively by the solver to guarantee the prescribed tolerances.

Relative tolerance: 10e-9

Absolute tolerance: 10e-10

Minimum step size: 10e-9s (1 ns).

#### Visualization Timescale

The following values were used for the temporal resolution of simulation and dynamic timescales for visualization. Note that these parameters are for visualization only and are not associated with the integration step in backend integrator.

Temporal resolution: 10 ms

Visualization rate (normal): 100 ms/s

Visualization rate (during transition or ablation): 40 ms/s.

#### Parameters for Neural Integration

The values of parameters for each connection described in Equation 2–6 are not precisely determined. However, we assume reasonable values reported in the literature (Wicks et al., 1996; Varshney et al., 2011). We assume each individual gap and synaptic junction has approximate conductance of g = 100 pS (Varshney et al., 2011), cell membrane conductance G^c^ = 10 pS, and membrane capacitance C = 1.5 pF (Varshney et al., 2011). We take leakage potential E_cell_ = −35 mV while reversal potential E_j = 0mV for excitatory synapses and −48 *mV* for inhibitory synapses (Wicks et al., 1996). For the synaptic activity variable, we take ${\text{a}}_{\text{r}}=\frac{1}{1.5}$, ${\text{a}}_{\text{d}}=\frac{5}{1.5}$ and width of the sigmoid β = 0.125mV^−1^ (Wicks et al., 1996). Also for the initial condition of the membrane voltages V and synaptic activity variable s, we sample the normal distribution of μ = 0 and σ = 0.94 with size 279 ^*^ 2 (for both V and s) and multiply by 10^−4^. To validate the simulation and the choice of parameters we tested for robustness by perturbing (±20%) individual connection strengths and each neuron's parameters, showing that dynamic functionality persists.

#### Parameters for Synchronization

**Figure 6:**
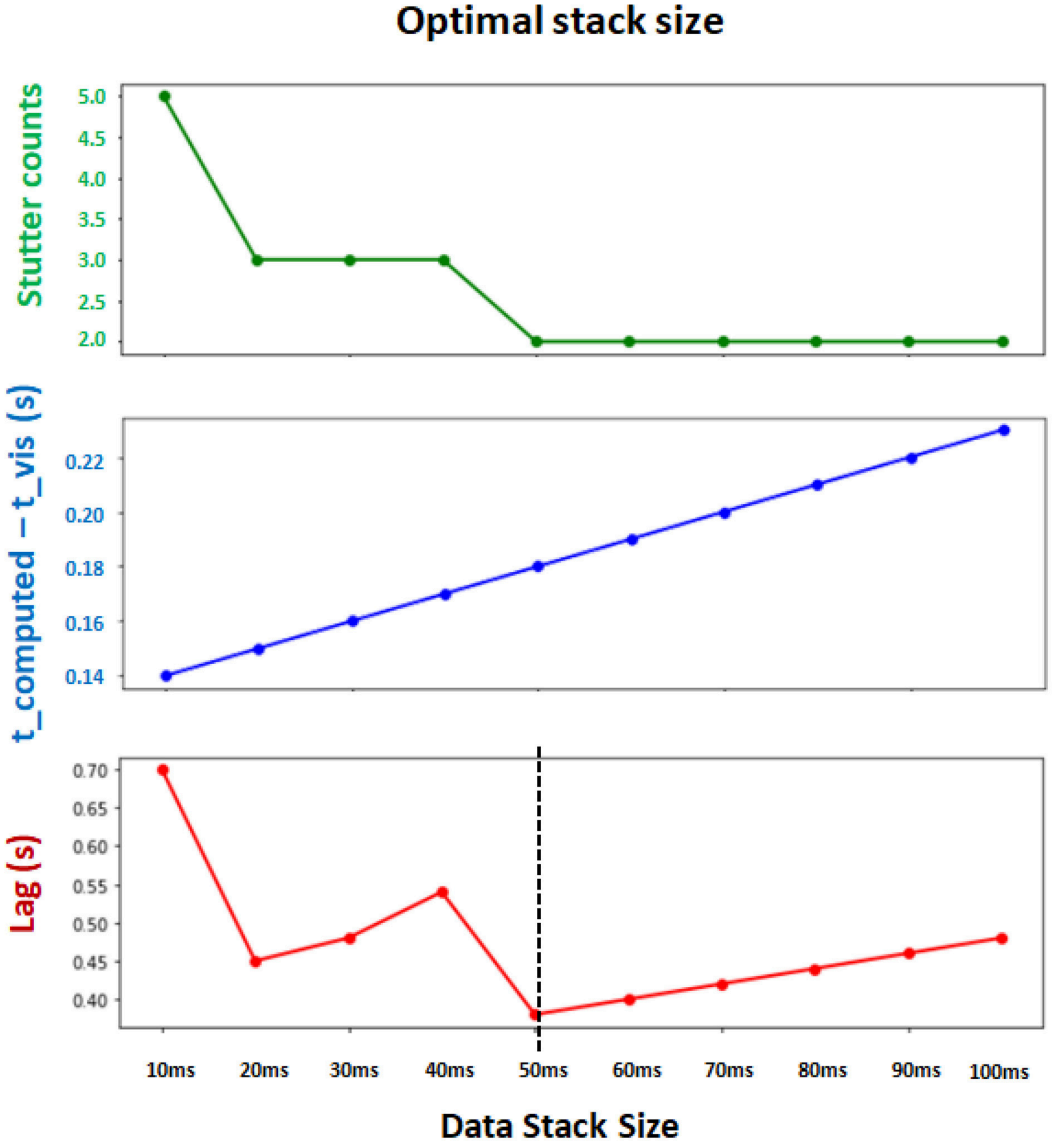
Determining the optimal Δt (data stack size) for synchronization and computational efficiency. Two evaluation metrics are collected for different choices of stack size. t_computed − t_vis measures the synchronization between the leading integration time point in backend and leading visualization time point in frontend (lower is better). Stutter counts represents the computational efficiency by counting the number of stutters (i.e., when visualization pauses with t_computed = t_vis; lower is better). Both metrics are measured with identical sessions of 10 s simulation. Final evaluation metric (Lag) is obtained by multiplying these two metrics (lower is better). The results show that Δt = 50 *ms* achieves the minimum lag, and supports the best balance between synchronization and computational efficiency.

The optimal values for Δ*t*, t_buffer_, and internal refractory period τ in Equation 9 depend on computing power of the system. We found the parameters Δt = 50 ms, t_buffer_ = 100 ms and τ = 50 ms (in actual time) to be of reasonable default values which achieve both computational efficiency and synchronization between the interface and backend (Figure 6). Note that Δt and t_buffer_ are in simulation timescale while τ is measured in computer's internal timer.

#### Parameters for Stimuli Transition

We use *t*_*offset*_ = 150 *ms*, and *r* = 0.025 in Equation 10. We found these values to be the optimal choice since the transition curve does not induce abrupt shift in *dynome* dynamics, and the visualization rate remains to be fast enough. Given the value of *r*, the time it takes for complete transition from one stimulus amplitude to the other is approximately 2*t*_*offset*_. Thus, for our choice of parameters for *C. elegans* simulations, the transitional period is around 300 ms.

#### Computation of Input Current Unit

From Equation 2–4 and physiological parameters specified above, the unit of input current is pS^*^mV = 10^−15^*A* = *fA* (femto-ampere). However, in our implementation, we divided both sides of Equation 2 by conductance constant 100 pS. This gives 1arb (arbitrary unit of input) = 10^−13^*A* = 0.1 *pA*, implying 1000 arb = 100 pA = 0.1 nA. We verified these units with the I-V curves measured in Goodman et al. (1998).

## Author Contributions

ES and JK conceptualized the framework. ES acquired the funding for the study. ES, JK, and WL developed the methodology of the work. JK and WL developed the software. JK and ES validated the results. JK and ES wrote the original draft of manuscript. JK and ES edited and revised the manuscript.

### Conflict of Interest Statement

The authors declare that the research was conducted in the absence of any commercial or financial relationships that could be construed as a potential conflict of interest.
